# Structural Factors of Preschoolers’ Creative Personality and Their Impact on Creative Thinking Based on the Componential Model of Creativity

**DOI:** 10.3390/bs16060971

**Published:** 2026-06-11

**Authors:** Nalanying Pulie, Chao Jin, Wen Liu, Liting Tan

**Affiliations:** 1 College of Psychology, Liaoning Normal University, 850 Huanghe Road, Sha Hekou District, Dalian 116029, China; psypl_913@lnnu.edu.cn (N.P.); psyjc101359@lnnu.edu.cn (C.J.); psyqqqhhh157@lnnu.edu.cn (L.T.); 2 School of Education, Taiyuan Normal University, Taiyuan 030619, China

**Keywords:** preschoolers, creative personality, creative thinking, componential model of creativity

## Abstract

Early childhood is a critical period for creative personality development. Guided by Amabile’s Componential Model of Creativity, this research explored the higher-order structure, developmental trajectories, and predictive role of preschoolers’ creative personality. Study 1 suggested an emergent three-factor higher-order structure comprising intrinsic driving, growing, and openness factors. Due to the longitudinal instability of the openness factor observed at this age, subsequent analyses focused on the two core components. Study 2 employed longitudinal latent growth modeling to examine the developmental trajectories of the intrinsic driving and growing factors across three waves. Study 3 assessed the predictive effects of these two factors on creative thinking performance (specifically figural divergent thinking). The results indicated that: (1) the nine teacher-rated dimensions of preschoolers’ creative personality exhibited preliminary evidence of multidimensional higher-order organization; (2) both intrinsic driving and growing factors significantly increased with age, following distinct linear trajectories; and (3) only the intrinsic driving factor significantly predicted figural divergent thinking in the structural model. While an emergent three-factor structure appeared in cross-sectional data, the longitudinal and predictive findings primarily support the stability and relevance of the core socio-motivational components. Teacher-observed personality tendencies are relevant to early figural divergent thinking but should not be interpreted as evidence for creativity as a whole. These results suggest the relevance of intrinsic driving tendencies to preschoolers’ figural divergent thinking.

## 1. Introduction

Creative personality is crucial for creativity (Lin, 2018). Early childhood is a critical period when divergent thinking surges at ages 4–6, and playfulness predicts later creativity (Bai et al., 2024; Fung & Chung, 2025). Early creative personality predicts later creativity (Gardner, 1999). A review of the past two decades of research on children’s creativity indicates that exploration from a creative personality perspective remains limited. In addition, previous studies on children’s creativity mostly focused on the primary and secondary school stage (6–18 years old), with relatively few examining preschoolers (3–6 years). Although recent studies have shown that family and educational contexts can support preschoolers’ creative tendencies (D. Shi et al., 2024; J. He et al., 2024; Fan et al., 2024), these findings mainly explain external conditions of creativity rather than the internal organization of preschoolers’ creative personality. What remains less clear is how different personality-related tendencies in early childhood are structured and whether these tendencies form broader higher-order factors that are meaningful for understanding creative development. Therefore, a more focused examination of preschoolers’ creative personality is needed, not only to identify relevant characteristics, but also to clarify whether these characteristics can be organized into theoretically meaningful higher-order dimensions.

### 1.1. Preschoolers’ Creative Personality

In early childhood, these personality-related tendencies should be understood as relatively plastic rather than fixed adult-like traits. This developmental plasticity makes the preschool years an appropriate period for examining how personality-related tendencies are organized.

Preschoolers’ creative personality is conceptualized as a set of relatively stable but developmentally evolving dispositional tendencies that support preschoolers’ engagement in creative activities (W. Liu & Qi, 2008; W. Liu & Zhang, 2021). This construct is treated as personality-related rather than as momentary motivation, observable behavior, or creative performance itself (Slobodskaya, 2021). Motivational features such as curiosity and interest are included only when they reflect relatively consistent tendencies across situations, whereas behaviors such as exploration, persistence, cooperation, and self-expression are regarded as observable manifestations of these underlying tendencies. In this sense, creative personality is also distinguished from creative thinking, which is examined in the present study as an outcome-related indicator of creativity. Researchers have examined the personality structure of Chinese preschoolers and identified five dimensions: intellectual characteristics, conscientiousness/self-control, extraversion, prosociality, and emotional stability (W. Liu & Yang, 1999; Gao et al., 2019). These findings provide a developmental background for studying preschool personality, but they do not clarify how creative personality itself may be organized at a higher-order level.

Operationally, the present study assessed preschoolers’ creative personality using the nine-dimensional framework developed by Liu and colleagues, including curiosity, sensitivity, novelty, self-confidence, cooperation, independence, achievement, aesthetics, and sense of humor (W. Liu et al., 2020; W. Liu & Zhang, 2021; W. Liu & Qi, 2008). These dimensions were treated as measurable indicators of broader dispositional tendencies in early childhood. They were not intended to define creative personality as a set of isolated behaviors, nor were they treated as equivalent to creative thinking or creative performance.

### 1.2. The Componential Model of Creativity

Examining the higher-order organization of personality-related characteristics may help clarify how broader dispositional patterns are associated with creativity. From the perspective of creative personality structure, scholars such as Li et al. (2014) argue that creative personality has a higher-order structure, which has a closer relationship with creativity than individual creative personality dimensions (Qian et al., 2010). However, while structured trait models have been studied in adolescent and adult creative personality, the structural features of preschoolers’ creative personality remain underexplored.

The present study draws on Amabile’s componential theory of creativity as a framework for organizing personality-related tendencies, rather than as a direct definition of creative personality. Amabile’s model proposes that creativity arises from the joint contribution of task motivation, creativity-relevant processes, and domain-relevant skills, all of which are shaped by the social environment (T. M. Amabile, 1983; T. M. Amabile, 1996; T. M. Amabile et al., 1986; T. Amabile, 2012). In early childhood, these components are still emerging and are expressed in age-appropriate forms (Kaufman & Beghetto, 2009). In particular, domain-relevant skills at this stage are not yet organized as stable, specialized bodies of knowledge or technical expertise comparable to those observed in older children, adolescents, or adults. Rather, they are embedded in everyday activities such as play, drawing, storytelling, movement, and exploratory problem solving, and are strongly shaped by children’s immediate experiences and learning opportunities (Ourda et al., 2025a, 2025b; Rafiei et al., 2025).

Although intrinsic motivation has long been regarded as central to creativity, externally shaped forms of motivation may also affect creative expression, especially in educational contexts (T. M. Amabile et al., 1986; T. Amabile, 2012). Accordingly, the present study does not examine task-specific motivational states in particular classroom situations. Instead, it focuses on broader teacher-rated dispositional characteristics that may include relatively stable motivational tendencies.

**Figure 1:**
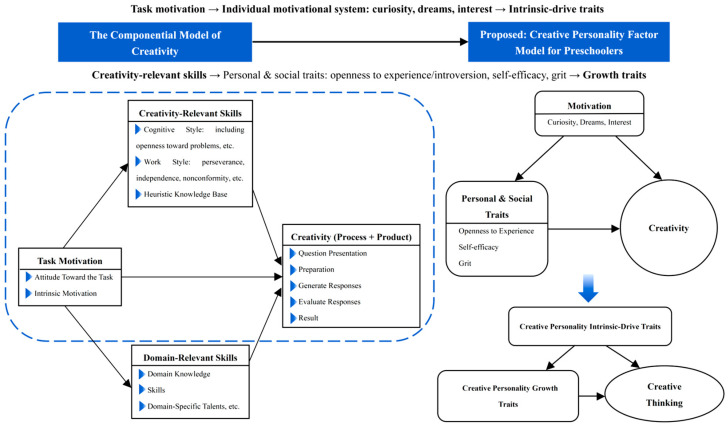
Flowchart of the construction process of the creative personality factor model for preschoolers.

Previous studies suggest that the nine dimensions of 3–6-year-olds’ creative personality may be organized into higher-order patterns, but this structure has not yet been firmly established in preschool samples. Drawing on Amabile’s Componential Model of Creativity and prior research on preschoolers’ creative personality, several alternative higher-order factor models were constructed and compared. The labels used in this study, including the intrinsic driving factor and the growing factor, are analytic and interpretive labels for the factor patterns observed in the present data. They should not be read as established developmental constructs, nor as evidence that preschoolers’ creative personality is organized in the same way across settings. In Amabile’s model, domain-relevant skills refer to knowledge, techniques, and abilities within a particular field. For preschoolers, however, such skills are still rudimentary, unevenly developed, and closely tied to specific activity contexts. They are more appropriately reflected in task-based creative performance or domain-specific assessments than in a general teacher-rated personality questionnaire. Therefore, the present framework focuses on two developmentally appropriate aspects that could be represented by the nine creative personality dimensions: relatively stable motivational tendencies and creativity-relevant processes. In this mapping, the intrinsic driving factor was interpreted as a pattern centered on curiosity, sensitivity, and novelty, whereas the growing factor was interpreted as a pattern involving self-confidence, cooperation, independence, and achievement (T. Amabile, 2012; Kaufman & Beghetto, 2009). Thus, the present framework should be understood as a developmental adaptation of the componential model for the study of preschoolers’ creative personality, rather than a full reproduction of all the components of the original theory. Domain-relevant skills remain theoretically important, but they were treated as outside the scope of the present personality-based measurement model. The model is shown in Figure 1.

#### 1.2.1. The Construction of Components and Their Relationship

The higher-order factor model was specified from the nine measured dimensions of preschoolers’ creative personality. From a developmental and componential perspective, these dimensions were expected to cluster around broader dispositional tendencies related to intrinsic engagement, creativity-relevant processes, and openness-related expressive characteristics. Specifically, intrinsic engagement reflects preschoolers’ attraction to novelty and exploration, whereas creativity-relevant processes refer to emerging patterns of confidence, autonomy, persistence, and socially supported engagement that help preschoolers approach and sustain creative activities. In the present model, the intrinsic driving factor was used to describe the clustering of curiosity, sensitivity, and novelty seeking. This pattern may indicate teachers’ observations of preschoolers’ relatively consistent interest in new ideas, materials, and activities (T. Amabile, 2012; Zhang et al., 2021). The growing factor was used to describe the clustering of self-confidence, cooperation, independence, and achievement, which may represent teacher-observed resources that help preschoolers participate in and sustain open-ended activities. In the present study, self-confidence reflects preschoolers’ willingness to try out ideas and tolerate uncertainty during open-ended activities; independence reflects an emerging tendency to approach problems autonomously and flexibly; achievement reflects persistence and task commitment when facing challenging activities; and cooperation reflects preschoolers’ capacity to participate in socially embedded creative tasks, respond to others’ ideas, and sustain engagement in shared problem solving. Thus, although these four dimensions include agentic, social, and self-regulatory features, they are grouped together because they represent age-appropriate expressions of creativity-relevant processes in early childhood rather than isolated social or motivational traits (T. Amabile, 2012; Qian et al., 2010).

In the present study, these terms are used as provisional descriptive labels for broader higher-order tendencies identified in the data and interpreted through Amabile’s theoretical distinction between motivational tendencies and creativity-relevant processes. These labels are neither fully established nor purely data-driven. Instead, they integrate empirical clustering of nine measured dimensions with Amabile’s theoretical distinction between motivational tendencies and creativity-relevant processes. The intrinsic driving factor summarizes a pattern centered on preschoolers’ attraction to novelty and exploratory engagement, whereas the growing factor summarizes a pattern centered on developing confidence, autonomy, persistence, and social engagement that may enable preschoolers to apply creativity-relevant processes in age-appropriate creative activities (W. Liu & Qi, 2008; Li et al., 2014; Qian et al., 2010).

The Componential Model of Creativity suggests that motivational tendencies and creativity-relevant processes jointly support creative expression. In the present study, this theoretical relation was translated into a developmental hypothesis: preschoolers’ intrinsic attraction to novelty and exploration may be associated with the emerging confidence, autonomy, persistence, and socially supported engagement captured by the growing factor. Therefore, the association between the intrinsic driving factor and the growing factor was examined empirically, rather than assumed as a direct replication of the adult-based componential model.

#### 1.2.2. Impact of Components on Creativity

The Componential Model of Creativity also helps explain why personality-related tendencies may be associated with creative outcomes. Although domain-relevant skills were not modeled as part of preschoolers’ creative personality, they may still contribute to preschoolers’ performance in specific creative tasks. In the present study, the outcome variable is creative thinking rather than creativity in a broad sense.

Prior findings on the relation between creative personality and creative outcomes have been mixed. One reason is that previous studies have differed in the age groups examined, the level at which personality was measured, and the type of creative outcome used. In studies with older children or adults, personality is often treated as a relatively stable trait system and linked to creative achievement or domain-specific performance. In early childhood, however, personality-related tendencies are still developing and may be expressed through everyday engagement, exploration, persistence, and social participation rather than through mature creative products (Kaufman & Beghetto, 2009). The findings may also vary because some studies examine isolated traits, whereas others examine broader personality profiles. Similarly, creative outcomes range from divergent-thinking tasks to teacher judgments, classroom products, and later creative achievement, making direct comparison difficult (Sternberg & Lubart, 1995; Zhang et al., 2021; Lebuda et al., 2025). The present study addresses this issue in a limited but more specific way. It defines preschoolers’ creative personality as teacher-rated dispositional tendencies, examines whether these tendencies can be represented by higher-order latent groupings, and tests their association with a specific figural divergent thinking task. Thus, the purpose is not to show that creative personality directly determines creativity as a whole. Rather, the study asks whether particular higher-order patterns in teacher-rated creative personality are associated with preschoolers’ performance on an age-appropriate measure of creative thinking.

Early childhood provides an appropriate period for examining this relation because personality-related tendencies and creative thinking both undergo rapid development during the preschool years (Bronfenbrenner & Morris, 2006; Kaufman & Beghetto, 2009; G. Liu et al., 2013). In drawing, storytelling, play, and exploratory problem solving, preschoolers’ curiosity, persistence, autonomy, and willingness to engage may influence the extent to which they generate diverse and original responses. Accordingly, creative thinking was used in the present study as an age-appropriate indicator of early creative potential. The intrinsic driving factor was hypothesized to exhibit a stronger positive association with figural divergent thinking. This expectation was based on the factor’s reflection of curiosity, sensitivity, and an attraction to novelty, traits closely linked to open-ended creative task engagement. The growing factor was also expected to be positively related to creative thinking, but its relation was expected to be less direct because it reflects self-regulatory and socially supported resources, such as confidence, autonomy, persistence, and cooperation, that help preschoolers sustain creative engagement.

### 1.3. Present Study

To address the above gaps, the present research combined three studies. Study 1 examined whether the nine teacher-rated creative personality tendencies could be organized into a meaningful higher-order structure in preschoolers. Study 2 explored how the teacher-perceived higher-order tendencies identified in Study 1 changed across the preschool years and their developmental trajectories. Study 3 examined whether these higher-order tendencies were related to preschoolers’ figural creative thinking performance, with particular attention to whether the intrinsic driving factor showed a stronger association with creative thinking than the growing factor. Across these three studies, the central contribution of the present research is to clarify a developmentally appropriate higher-order account of preschoolers’ creative personality as perceived in the classroom. Rather than treating the three studies as separate lines of evidence, the analysis follows a single sequence: identifying the emergent higher-order structure, examining the developmental course of the focal factors, and testing their relevance for specifically measured figural creative thinking. Based on these objectives, the hypotheses of the present research are as follows:

**Hypothesis** **1.** *The nine teacher-rated measured dimensions of preschoolers’ creative personality would be organized into a theoretically interpretable emergent higher-order structure, with curiosity, sensitivity, and novelty forming an intrinsic driving factor; self-confidence, cooperation, independence, and achievement forming a growing factor; and aesthetics and sense of humor forming an openness-related factor.*

**Hypothesis** **2.** *The scores of intrinsic driving and growing factors would increase across the preschool years, reflecting maturational trends in perceived motivational tendencies and creativity-relevant processes. Individual differences were expected to appear in both initial levels and rates of change, especially for the intrinsic driving factor.*

**Hypothesis** **3.** *The intrinsic driving and growing factors would be positively associated at the initial level, and their developmental trajectories would also be related, reflecting a co-developmental trend between observed motivational tendencies and creativity-relevant processes.*

**Hypothesis** **4.** *The intrinsic driving factor would be positively associated with preschoolers’ figural divergent thinking performance and was expected to show a stronger association than the growing factor. The growing factor was also expected to be positively related to figural task performance, but its relation was expected to be less direct because it reflects self-regulatory and social resources that support sustained creative engagement.*

## 2. Study 1: The Exploration of Components of Preschoolers’ Creative Personality

### 2.1. Methods

#### 2.1.1. Participants

This study was conducted with 720 preschoolers (aged 3–6 years) recruited from an urban public preschool. Nineteen invalid data points (those who did not complete the test) were excluded, and 701 valid data points were obtained. Detailed participant characteristics are presented in Table 1. The sample included 30 six-year-olds (4.3% of the total sample). Although this group is smaller than others due to school-readiness transitions, this group has been retained to represent the entire preschool age range. However, its uneven distribution constitutes a significant limitation. Consequently, any age-related comparisons or interpretations involving the 6-year-old cohort should be regarded as preliminary and exploratory. The overall factor stability in Study 1, however, remains robust due to the large total sample size. Independent-samples *t*-tests were conducted to examine potential differences between valid and invalid participants. The results showed no significant differences on key demographic variables or on any of the nine creative personality dimensions (*p*s > 0.05, Cohen’s d ranging from 0.03 to 0.10, 95% CIs [−0.16, 0.29]). The effect sizes for these differences were negligible, suggesting that participant exclusion did not introduce systematic bias. This study was approved by the Ethics Committee of Liaoning Normal University, and informed consent forms were signed by all the participants’ teachers and guardians.

#### 2.1.2. Measures

##### Preschoolers’ Creative Personality Questionnaire

This study used the teacher-rated questionnaire developed by Liu et al. (W. Liu & Qi, 2008; W. Liu & Zhang, 2021). The 44-item instrument employs a 5-point Likert scale (1 = strongly disagree to 5 = strongly agree). Cronbach’s α reliability in the current sample was 0.94. Before data collection, all the teacher-raters underwent standardized training to facilitate a consistent understanding of the creative personality dimensions. To minimize expectancy bias, teachers remained blind to the study’s hypotheses and the preschoolers’ performance on other tasks. In cases of significant disagreement (e.g., a discrepancy of more than 1 point), raters resolved the difference through consensus-building discussions; otherwise, the average of the two ratings was used.

#### 2.1.3. Procedure

Following informed consent from teachers and guardians, two teachers independently rated each class. Inter-rater reliability was assessed using the intraclass correlation coefficient (ICC) based on a two-way random effects model, absolute agreement, and average measures (ICC [2, k]). The ICC values for all the subscales ranged from 0.75 to 0.88 (95% CI [0.77, 0.92]), indicating good to excellent consistency according to the criteria proposed by Koo and Li (2016). Final scores represented the average of both ratings.

#### 2.1.4. Data Analysis

Data management and analysis utilized Microsoft Excel, SPSS 26.0, Mplus 8.0 and MS SQL Server 18.4. The measurement tools employed in this study were all teacher-reported questionnaires, which may introduce common method bias. Consequently, Harman’s single-factor test was conducted to assess the presence of significant common method bias (Podsakoff et al., 2003; Tang & Wen, 2020). The first principal component explained 28.90% of the total variance, which was below the 40% criterion, indicating no significant common method bias. This study employed an item-to-construct parceling strategy by aggregating the 44 items into nine dimensions, and level mean scores for the initial exploratory analysis. According to Little et al. (2002), this approach is appropriate when the primary interest lies in the relations among latent constructs rather than the items themselves, as it reduces random error and improves the model’s parsimony. To facilitate the validity of this aggregation, item-level CFA was subsequently conducted to verify the structure at the granular level. The higher-order factor structure was evaluated using the total sample. Given that the majority of the sample consisted of preschoolers aged 3–5, the resulting model primarily represents the structural characteristics of the creative personality during the core preschool years. While the 6-year-old cohort was included to maintain the developmental scope of the study, the stability of this specific structure for preschoolers transitioning to primary school remains exploratory.

### 2.2. Results

#### 2.2.1. Explore Factor Structure

Exploratory Factor Analysis (EFA) was conducted on the nine-dimensional mean scores. This parceling approach was employed to maintain a favorable variable-to-sample ratio and focus on the higher-order integration of the dimensions, and to maintain conceptual consistency with the established nine-dimensional framework of the Preschoolers’ Creative Personality Questionnaire (W. Liu & Qi, 2008; W. Liu & Zhang, 2021). Exploratory factor analysis of the nine-dimensional mean scores indicated adequate sampling (KMO = 0.88) and significant sphericity (Bartlett’s χ^2^(36) = 5922.47, *p* < 0.001). As an initial exploratory step, exploratory factor analysis with Promax rotation identified five factors (Curiosity, Sensitivity, Novelty, Self-Confidence, and Cooperation) explaining 68.83% of the variance. These preliminary findings, along with Amabile’s theoretical framework, informed the construction of the subsequent competing higher-order models to identify a more theoretically parsimonious structure.

#### 2.2.2. Competing Higher-Order Models

Six competing higher-order models were specified based on Amabile’s Componential theory of creativity (As Table 2). Grounded in Amabile’s componential theory of creativity, this study developed six competing models centered on two primary higher-order models. Model 1 was derived directly from the initial EFA structure, while Model 2 expanded the growing factor by incorporating independence and achievement based on their conceptual definitions. Models 3 through 6 then systematically examined the structural placement of aesthetics and sense of humor, testing whether these dimensions should be integrated into the pre-established factors or function as an independent higher-order dimension (Table 2).

Model fit was evaluated using multiple indices (χ^2^, df, CFI, TLI, RMSEA, SRMR, AIC, and BIC, in Table 3). Although Models 1 and 2 exhibited the best absolute fit indices, this advantage was primarily attributable to their greater parsimony (i.e., fewer factors and higher degrees of freedom), which tends to produce artificially favorable fit statistics in non-nested model comparisons. Model 6 was excluded first due to unacceptable overall fit. Although Models 3 and 4 showed acceptable fit, Model 5 provided a clearer theoretical differentiation among intrinsic driving, growing, and openness-related factors. It achieved an acceptable-to-good fit while offering the strongest theoretical correspondence with the componential model by clearly separating three higher-order factors (intrinsic driving, growing, and openness).

Consequently, Model 5 was selected as the most appropriate higher-order structure among the tested candidates, as it best reconciled statistical evidence with theoretical expectations regarding the multidimensional nature of preschoolers’ creative personality.

#### 2.2.3. Second-Order CFA Assessment

**Figure 2:**
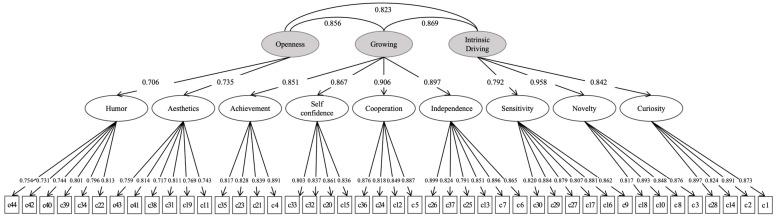
Higher-order confirmatory factor model of preschoolers’ creative personality.

Confirmatory factor analysis (CFA) was conducted on all 44 items. The first-order model indicated acceptable fit: χ^2^(866) = 1572.12, CFI = 0.94, TLI = 0.92, RMSEA = 0.07 (90% CI [0.06, 0.08]), SRMR = 0.06. The second-order model, consisting of three latent factors—intrinsic driving, growing, and openness—also achieved good fit: χ^2^(890) = 1731.09, CFI = 0.92, TLI = 0.91, RMSEA = 0.07 (90% CI [0.06, 0.07]), SRMR = 0.06. All the standardized second-order factor loadings were relatively large and statistically significant (*p*s < 0.001), ranging from 0.70 to 0.89 (SEs = 0.02–0.04; 95% CIs [0.67, 0.98]). These loadings indicated substantial associations, with the higher-order factors explaining 49% to 79% of the variance (R^2^) in the first-order dimensions (Figure 2). From a measurement perspective, these R^2^ values suggest that the higher-order factors accounted for a substantial proportion of variance in the first-order dimensions, providing preliminary support for the higher-order representation.

#### 2.2.4. Reliability

The measurements demonstrated robust internal consistency. The overall Cronbach’s α was 0.86 (95% CI [0.84, 0.88]). For the higher-order factors, the reliability coefficients were as follows: intrinsic driving factor, α = 0.79 (95% CI [0.76, 0.82]); growing factor, α = 0.79 (95% CI [0.76, 0.82]); openness factor, α = 0.83 (95% CI [0.80, 0.86]). The nine individual dimensions also exhibited acceptable reliability, with α values ranging from 0.74 to 0.87 (95% CIs [0.70, 0.89]). These results indicate that the creative personality constructs were measured with sufficient precision across all dimensions.

## 3. Study 2: The Internal Relationships Among Components of Preschoolers’ Creative Personality

Although Study 1 suggested an emergent three-factor higher-order structure, providing an initial exploratory framework for understanding preschoolers’ creative dispositions, only the intrinsic driving and growing factors were carried forward to the longitudinal (Study 2) and predictive (Study 3) analyses. This decision was informed by both the empirical findings and theoretical considerations. In the longitudinal sample (N = 142), the openness factor (aesthetics and sense of humor) suggested relatively low internal consistency (Cronbach’s α = 0.58 at T1, 0.61 at T2, and 0.55 at T3) and modest test–retest reliability across the three waves (*r* = 0.29 from T1 to T2; *r* = 0.34 from T2 to T3). Furthermore, attempting to include all three higher-order factors in the latent growth curve models resulted in non-convergence and improper solutions due to over-parameterization relative to the sample size. From a developmental perspective, while motivational and self-regulatory aspects of creativity appear relatively stable in preschoolers, aesthetic appreciation and sense of humor exhibit distinctive and still-maturing characteristics during the preschool period (Huang et al., 2020; W. Liu, 2019). Consequently, Studies 2 and 3 should be interpreted as analyses of two focal higher-order groupings rather than as longitudinal or predictive validation of the complete three-factor model identified in Study 1.

### 3.1. Methods

#### 3.1.1. Participants

A three-year longitudinal study was conducted with 160 preschoolers (aged 3–6 years) recruited from an urban public preschool. After accounting for attrition, 142 participants provided valid data across the study period. While this sample size is modest for SEM, it was deemed adequate for the current models for several reasons. First, the models were kept parsimonious by focusing on the two primary higher-order factors and utilizing dimension-level parcels, maintaining a favorable sample size-to-parameter ratio (Kline, 2016). Second, simulation studies suggest that for longitudinal models with high factor loadings (as seen in the data, >0.70), sample sizes of N > 100 can provide sufficient power and stable estimates (Wolf et al., 2013). Third, the use of three waves of data with low attrition helps to robustly estimate the linear growth parameters.

Additionally, 34 female head teachers rated children’s creative personality. Independent-samples *t*-tests revealed no significant differences between included and excluded participants (*p*s > 0.05). The effect sizes for these differences were negligible, with Cohen’s d ranging from 0.04 to 0.13, suggesting that dropout was not systematic and did not bias the longitudinal results. Detailed participant characteristics are presented in Table 4. This study was approved by the Ethics Committee of Liaoning Normal University, and informed consent forms were signed by all the participants’ teachers and guardians.

#### 3.1.2. Measures

##### Preschoolers’ Creative Personality Questionnaire

The questionnaire was used the same as in Study 1.

#### 3.1.3. Procedure

Following informed consent from teachers and guardians, assessments were conducted annually over three years. Two teachers independently rated each class. Inter-rater reliability was assessed using the intraclass correlation coefficient (ICC) based on a two-way random effects model, absolute agreement, and average measures (ICC [2, k]). The ICC values for all the subscales and time points ranged from 0.76 to 0.87 (95% CI [0.73, 0.93]), indicating good to excellent consistency according to the criteria proposed by Koo and Li (2016). The final scores represented the average of both ratings.

#### 3.1.4. Data Analysis

Data management and analysis utilized Microsoft Excel, SPSS 26.0, Mplus 8.0 and MS SQL Server 18.4. Analytical procedures included the latent variable linear growth model based on structural equation modeling.

The measurement tools employed in this study were all the teacher-reported questionnaires, which may introduce common method bias. Consequently, Harman’s single-factor test was conducted to assess the presence of significant common method bias (Podsakoff et al., 2003; Tang & Wen, 2020). The results showed that the first principal component explained 29.14% of the total variance at T1, 31.43% at T2, and 32.79% at T3, with the variance explained at each time point remaining below 40%, confirming the absence of significant common method bias in this study’s measurements.

#### 3.1.5. Missing Data Analysis

Due to the longitudinal design, there was participant attrition across the three waves. All 160 participants completed the T1 assessment and were included in subsequent analyses. Attrition rates were 7.5% at T2 (n = 148) and 11.25% at T3 (n = 142). Attrition analyses were conducted to assess potential selective dropout by comparing participants with complete versus incomplete data across waves. Independent-samples *t*-tests showed no significant differences between retained and attrited participants on key demographic variables (age and gender) or on any of the nine creative personality dimensions (*p*s > 0.05, Cohen’s d ranging from 0.02 to 0.14). The chi-square test also indicated no significant difference in sex ratio between the included and excluded participants, χ^2^(1) = 0.09, *p* = 0.76. Little’s test of missing completely at random (MCAR) was non-significant, χ^2^(344) = 372.15, *p* = 0.12, Cramér’s V = 0.01. Missing data were handled using full information maximum likelihood estimation (FIML), which uses all the available data without imputation and provides unbiased estimates under the assumption of data missing at random (Dong & Peng, 2013).

### 3.2. Results

#### 3.2.1. Descriptive Statistics

Descriptive statistics for preschoolers’ intrinsic driving factor and growing factor at age three are presented in Table 5.

#### 3.2.2. Longitudinal Measurement Invariance

The two-factor higher-order model (intrinsic driving and growing factors) indicated scalar invariance across the three waves (T1, T2, and T3), supporting latent mean comparisons (Table 6). The changes in CFI and TLI between the configural, metric, and scalar models were all within the recommended threshold of 0.01 (Cheung & Rensvold, 2002). However, when residual variances were constrained to be equal (Strict Invariance), the model fit showed a significant decline.

The results indicate that the two-factor higher-order structure of preschoolers’ creative personality is longitudinally invariant at the scalar level across the three waves, allowing meaningful comparisons of latent means over time. Strict invariance was not fully supported, suggesting minor differences in item residual variances across waves.

#### 3.2.3. Unconditional Latent Growth Linear Model for Intrinsic Driving Factor and Growing Factor

To evaluate the developmental trajectories, unconditional linear latent growth models were conducted. The model fit indices for both factors indicated a good fit to the data (As Table 7). For the model, the R^2^ values of 0.33 to 0.38 reflect a medium-to-large effect in longitudinal behavioral research. This indicates that linear growth trajectories account for approximately one-third of the individual differences in the developmental changes in creative personality across three years, demonstrating considerable practical utility in modeling the maturation process during the critical preschool period.

Table 8 presents the parameter estimates. Both factors showed significant initial levels (Intercept Means = 2.89 and 2.98, SEs = 0.02, 95% CIs [2.85, 2.93] and [2.94, 3.02], respectively, *p*s < 0.001) and significant linear increases over time (Slope Means = 0.21, SEs = 0.02, 95% CIs [0.17, 0.25], *p*s < 0.001).

Significant intercept variances were found for both the intrinsic driving factor (σ^2^ = 0.22, SE = 0.04, 95% CI [0.14, 0.30], *p* < 0.001) and the growing factor (σ^2^ = 0.09, SE = 0.03, 95% CI [0.03, 0.15], *p* < 0.001). These results indicate substantial individual differences in creative personality levels at age three (the baseline), providing a necessary statistical foundation for examining heterogeneous growth trajectories.

For the intrinsic driving factor, the slope variance was significant (σ^2^ = 0.05, 95% CI [0.01, 0.09], *p* = 0.01), indicating individual differences in the growth rate. A significant negative correlation between the intercept and slope (*r* = −0.31, *p* < 0.001, 95% CI [−0.51, −0.11]) suggested that preschoolers with lower initial levels of intrinsic driving tended to show faster increases over time. For the growing factor, however, the slope variance was not significant (σ^2^ = 0.01, 95% CI [−0.03, 0.05], *p* = 0.62), and the intercept–slope correlation was negligible (*r* = 0.03, 95% CI [−0.22, 0.28], *p* = 0.72), suggesting a relatively uniform growth rate among participants regardless of their initial status.

#### 3.2.4. Unconditional Parallel Latent Variable Linear Growth Model of Intrinsic Driving Factor and Growing Factor

**Figure 3:**
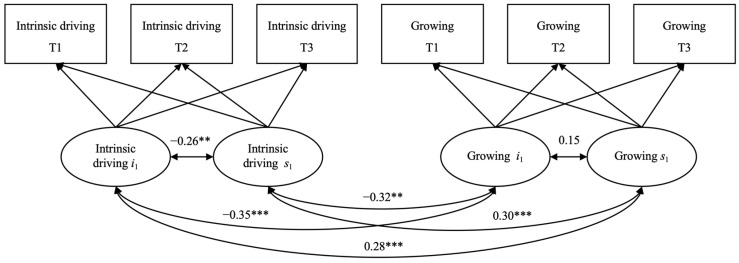
Unconditional parallel latent variable linear model of intrinsic driving factor and growing factor. Note: *** *p* < 0.001, ** *p* < 0.01.

The unconditional parallel latent linear growth model for intrinsic driving factor and growing factor indicated a good fit to the data, χ^2^(2) = 6.79, CFI = 0.96, TLI = 0.92, RMSEA = 0.03 (90% CI [0.01, 0.08]), SRMR = 0.01. The correlation estimates between the two factors are shown in Table 9. The parallel linear growth model of the two factors is shown in Figure 3.

Table 9 revealed meaningful developmental patterns. First, a significant positive correlation was found between the intercepts of the two factors (*r* = 0.35, *p* < 0.001, 95% CI [0.25, 0.49]). Second, a significant positive correlation existed between the slopes of the two factors (*r* = 0.30, *p* < 0.001, 95% CI [0.14, 0.44]). Third, the intrinsic driving factor’s intercept was significantly and negatively correlated with its own slope (*r* = −0.26, *p* = 0.002, 95% CI [−0.41, −0.10]), representing a compensation effect where preschoolers starting with lower levels of drive showed faster growth rates. In contrast, the growth rate of the growing factor was relatively uniform across participants, as its intercept and slope were not significantly correlated (*r* = 0.15, 95% CI [−0.02, 0.31], *p* = 0.07).

## 4. Study 3: The Influence of Creative Personality Components on Preschoolers’ Creative Thinking

To ensure conceptual precision, it is important to clarify the scope of the outcome measure. In this study, creative thinking is operationalized specifically as figural divergent thinking potential. By focusing on fluency, flexibility, and originality within a figural task, aim to capture a critical cognitive facet of creativity. Therefore, the findings of this study pertain specifically to figural divergent thinking and should not be generalized to other dimensions of creativity, such as verbal divergent thinking, social creativity, or everyday creative behavior.

### 4.1. Methods

#### 4.1.1. Participants

A total of 180 participants (aged 4–6 years) were recruited from an urban public preschool. Ten participants (4 boys and 6 girls) who had not completed the test were excluded. The final sample consisted of 170 preschoolers, including 91 boys (*M* = 5.00 years, *SD* = 0.72) and 79 girls (*M* = 4.90 years, *SD* = 0.71). Additionally, 34 female head teachers (all with ≥2 years of teaching experience) participated in all three waves of data collection. Independent-samples *t*-tests revealed no significant differences between included and excluded participants (*p*s > 0.05). The effect sizes were negligible (Cohen’s d = 0.12), suggesting that the exclusion of participants did not introduce systematic bias to the predictive model. This study was approved by the Ethics Committee of Liaoning Normal University, and informed consent forms were signed by all the participants’ teachers and guardians.

#### 4.1.2. Measures

##### Preschoolers’ Creative Personality Questionnaire

The questionnaire was used the same as in Study 1.

##### Torrance Tests of Creative Thinking (TTCT)

Creative thinking was operationalized as figural divergent thinking and assessed using the Parallel Lines Task from the Torrance Tests of Creative Thinking (TTCT) Figural Form (Torrance, 1998). This task was selected because it is suitable for preschoolers and reduces reliance on linguistic ability (Kim, 2006). In this figural divergent thinking task, preschoolers were asked to draw as many different pictures as possible by incorporating pairs of parallel lines provided on the stimulus sheet. The task was administered individually in a quiet room. Instructions were translated into Chinese and slightly simplified to facilitate clear comprehension by preschoolers.

The TTCT Parallel Lines Task has been successfully adapted and applied in several previous studies with Chinese preschoolers, demonstrating acceptable cultural applicability in this context (R. Peng et al., 2021; D. Peng et al., 2018; B. Shi et al., 2017). Following these studies, the standard TTCT scoring criteria were used, but raw scores were relied upon rather than U.S. normative scores to avoid potential cultural bias. The task was scored for fluency, flexibility, and originality. Fluency was assessed by counting the number of valid drawings, with no points awarded for repetitions. Flexibility was scored based on the number of valid drawings that fell into distinct conceptual categories defined in the TTCT scoring manual, with each unique category receiving one point. Originality was assessed by assigning one point to each valid drawing that did not belong to any category specified in the manual. All the tests were independently scored by two postgraduate students majoring in psychology. A paired-sample *t*-test showed no significant differences between the two raters’ scores (*p*s > 0.05). The final score was calculated as the average of the two raters’ evaluations.

#### 4.1.3. Procedure

The Preschoolers’ Creative Personality Questionnaire was administered first, following the same procedure as in Study 1. The ICC values for all the subscales ranged from 0.78 to 0.89 (95% CI [0.76, 0.93]), indicating good to excellent consistency according to the criteria proposed by Koo and Li (2016). Subsequently, the TTCT was conducted in a quiet classroom within the preschool. Two research assistants (postgraduate) were trained to test preschoolers. The test included two phases: a preparation phase and a formal testing phase. During the preparation phase, the assistants explained the drawing task requirements and indicated how to complete the task. They ensured that each preschooler fully understood the instructions before proceeding to the formal test. The formal testing phase lasted for 10 min, during which preschoolers completed 12 unfinished line-drawing tasks. The administration and scoring procedures strictly followed the TTCT Figural Form manual (Torrance, 1998).

#### 4.1.4. Data Analysis

Data management and analysis utilized Microsoft Excel, SPSS 26.0, and MS SQL Server 18.4. Analytical procedures included correlation analysis and latent variable regression models.

### 4.2. Results

#### 4.2.1. Descriptive Statistics and Correlation Analysis of Preschoolers’ Creative Thinking

Descriptive statistics for creative thinking are presented in Table 10. The correlation analysis results (Table 11) indicated that the intrinsic driving factor was significantly correlated with Fluency, Flexibility, and Originality, and the growing factor was significantly correlated with Flexibility.

#### 4.2.2. Structural Regression Model Within the Structural Equation Modeling (SEM)

Within the SEM framework, a structural regression model was used to test whether two latent creative personality factors predicted this latent figural creative thinking construct. In this model, the intrinsic driving factor was defined as a teacher-rated dispositional factor indexed by curiosity, novelty, and sensitivity, rather than as a direct measure of preschoolers’ motivational states. Age and gender were included as covariates to control for their potential effects on figural creative thinking.

The model provided a good overall fit, χ^2^(5) = 8.92, CFI = 0.97, TLI = 0.95, RMSEA = 0.07 (90% CI [0.01, 0.13]), SRMR = 0.06. All three indicators loaded significantly and positively on creative thinking (*p*s < 0.001), with standardized loadings of 0.95 (SE = 0.04, 95% CI [0.87, 1.00]), 0.89 (SE = 0.04, 95% CI [0.81, 0.97]), and 0.81 (SE = 0.04, 95% CI [0.73, 0.89]) for fluency, flexibility, and originality, respectively, indicating that the measurement model was reliable. The R^2^ values for these indicators ranged from 0.66 to 0.90, indicating the measurement model’s high reliability.

**Figure 4:**
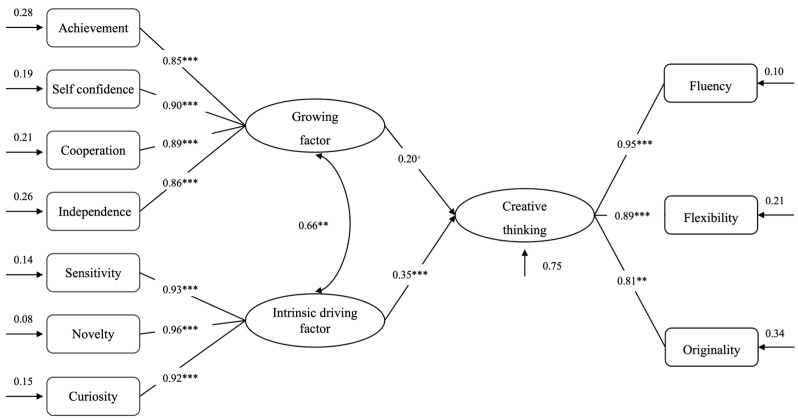
Regression model between creative personality and creative thinking. Note: *** *p* < 0.001, ** *p* < 0.01.

At the structural level (Figure 4), the model accounted for 25% of the total variance (R^2^ = 0.25) in preschoolers’ creative thinking. This level of explained variance suggests that the two teacher-rated creative personality factors accounted for a meaningful portion of individual differences in figural divergent thinking. Specifically, the intrinsic driving factor significantly and positively predicted creative thinking (β = 0.35, SE = 0.13, *p* = 0.007, 95% CI [0.10, 0.60]). In contrast, the growing factor did not significantly predict creative thinking (β = 0.20, SE = 0.12, *p* = 0.09, 95% CI [−0.04, 0.44]). Given the moderate correlation between the two predictors (*r* = 0.66), multicollinearity diagnostics were conducted. The Variance Inflation Factors (VIF) were 2.18 for intrinsic driving and 2.15 for the growing factor (Tolerance = 0.46 and 0.47, respectively), all within the acceptable threshold of 5.0 (O’Brien, 2007). Furthermore, a commonality analysis revealed that the unique contribution of intrinsic driving was ΔR^2^ = 0.07, while the growing factor contributed ΔR^2^ = 0.02, with 0.16 representing shared variance. This indicates that while both factors are theoretically and empirically related, the intrinsic driving factor serves as a more direct and cognitive engine, subsuming much of the predictive power that the growing factor exhibits at the bivariate level. This unique-versus-shared variance distribution suggests a potential practical consideration for educators: when aiming to support figural divergent thinking, it may be beneficial to consider the role of the intrinsic driving factor. The current findings suggest that this factor is more specifically associated with figural ideational performance in early childhood, while the growing factor may serve as a broader but less direct foundation for these specific cognitive tasks.

## 5. Discussion

The present study yielded three main findings. First, in cross-sectional analyses, the nine teacher-rated dimensions of preschoolers’ creative personality showed preliminary evidence of a three-factor higher-order organization consisting of intrinsic driving, growing, and openness factors. However, due to the longitudinal instability of the openness factor, subsequent analyses focused primarily on the two core factors: intrinsic driving and growing. Second, the intrinsic driving and growing factors showed mean-level increases across the preschool years. Third, the intrinsic driving factor was significantly associated with preschoolers’ performance on a figural divergent-thinking task, whereas the growing factor showed only a weaker and nonsignificant association. The discussion below focuses on the developmental meaning of these findings and on the limits of interpreting teacher-rated creative personality tendencies in relation to specific figural outcomes.

First, regarding the components of teacher-rated creative personality. Study 1 proposed an emergent three-factor structure, and subsequently provided longitudinal and predictive evidence for its two core components. Study 1 suggests that preschoolers’ creative personality tendencies, as perceived by teachers, were not simply a list of nine separate characteristics, but could be organized into broader teacher-observed tendencies. The intrinsic driving factor, defined by curiosity, novelty, and sensitivity, appears to reflect preschoolers’ tendency to notice, seek, and respond to new possibilities in classroom activities from the teachers’ perspective. This interpretation is close to the motivational side of the componential model, but it should be understood developmentally: the factor captures teachers’ observations of relatively consistent exploratory engagement, rather than preschoolers’ momentary motivational states. The growing factor, defined by self-confidence, cooperation, independence, and achievement, may be better understood as a set of emerging resources that teachers perceive as helping preschoolers sustain and share creative activity. In classroom contexts, these tendencies may be reflected in preschoolers’ observed willingness to try uncertain tasks, persist after difficulty, work with peers, and explain or revise their ideas. The openness factor, represented by aesthetic appreciation and sense of humor, was identified in the higher-order model but was not carried forward into the longitudinal and predictive analyses. This decision was mainly due to its limited psychometric stability in the longitudinal sample and the risk of over-parameterization in the latent growth models. Thus, the present study did not examine the full three-factor structure across all three studies, and the openness factor should be interpreted with caution (Bariaud, 1988; Huang et al., 2020; W. Liu, 2019; Shatskaya et al., 2024). Consequently, although Study 1 provided a structural representation of the openness factor at a single time point, its developmental trajectory and potential association with creative potential remain to be fully examined in longitudinal designs. (Kaufman & Beghetto, 2009).

Second, since Study 2 only analyzed intrinsic driving and growing, these longitudinal and predictive findings specifically apply to these two core teacher-rated components (intrinsic driving and growing) of the creative personality, rather than the comprehensive structure. This study found mean-level increases in both the intrinsic driving and growing factors across the three annual assessments, indicating that the teacher-rated tendencies represented by these two factors became more evident over time. Here, the intrinsic driving factor refers to curiosity-, novelty-, and sensitivity-related tendencies rated by teachers, rather than a direct measure of actual motivation (W. Liu & Yang, 2007; W. Liu & Zhang, 2021; W. Liu & Qi, 2008). These results are better read as evidence of average growth in rated scores, rather than as strong evidence that preschoolers followed clearly different developmental paths. The slope variance was significant but small for the intrinsic driving factor and nonsignificant for the growing factor, indicating that the present data detected limited individual differences in rates of change, especially for the growing factor. With only three waves, the analyses could test a basic linear trend but could not capture nonlinear or more complex developmental patterns. A critical observation from the parallel growth modeling is that while both factors increased over time, they followed largely distinct trajectories. In mature creative performance, as conceptualized in T. M. Amabile’s (1983) framework, motivational tendencies and social-regulatory skills are often described as highly integrated and functionally synergistic. However, longitudinal evidence limited to this specific developmental window reveals a pattern of parallel developmental change rather than functional coupling. Caution is required when interpreting these developmental findings. Instead, these findings only indicate that the structural integration of these perceived personality components may still be in an emergent phase during early childhood. Future research employing a lifespan perspective is necessary to determine if and when these parallel trajectories begin to converge into the more integrated systems observed in later stages of creative maturity.

Third, Study 3 examined how the two teacher-rated intrinsic driving and growing factors relate to figural divergent thinking performance in the Parallel Lines Task. The findings showed that the intrinsic driving factor was significantly associated with preschoolers’ task performance. This result is broadly consistent with classical motivational theories (Eisenberger & Shanock, 2003; Hennessey, 2019) and recent studies in early childhood (Nurjanah et al., 2024; Vaisarova et al., 2024) suggesting that characteristics related to intrinsic driving may be important for preschoolers’ creative engagement. In contrast, while the growing factor was positively associated with figural divergent thinking indicators in the bivariate correlations, its predictive effect in the SEM model did not reach statistical significance. This discrepancy likely results from the high correlation between the two factors (*r* = 0.66), which suggests a substantial overlap in the variance they explain. When both factors were entered into the structural model, the intrinsic driving factor accounted for more unique variance in figural divergent-thinking scores, whereas the growing factor showed limited incremental contribution. This nonsignificant result should not be treated simply as a failed prediction. The growing factor may capture classroom resources such as confidence, cooperation, independence, and achievement orientation, which teachers perceive as helping preschoolers participate in and sustain creative activities. However, these characteristics may not be directly associated with higher scores on a brief individual figural divergent-thinking task (Runco & Acar, 2012). To remain conservative regarding non-significant findings, it is suggested that the growing factor may become more influential during the school-age period, when preschoolers face increased demands for self-discipline and goal-directed persistence. This pattern is consistent with the theoretical model proposed by He and Chiang (W. He & Chiang, 2024), which suggests that growth in a creative mindset influences creative thinking primarily through the mediating role of creativity motivation. From a practical perspective, the findings tentatively suggest that educational settings supporting curiosity, exploration, and flexible responding may be relevant to preschoolers’ early creative expression. Teachers could potentially provide open-ended materials, such as blocks, loose parts, drawing tools, natural objects, and story cards, and invite preschoolers to generate more than one possible answer. In drawing, construction, pretend play, and storytelling, teachers may give preschoolers time to try, revise, and explain unusual ideas, rather than evaluating only the final product. These suggestions are speculative practical implications drawn from correlational findings rather than evidence-based prescriptions from an intervention study.

In the present study, the outcome was limited to figural divergent thinking, assessed through fluency, flexibility, and originality (Bai et al., 2024). These indicators provided the basis for the estimate of the creative potential measure used here. However, the current findings do not justify treating them as the full set of core dimensions of preschoolers’ creative thinking more generally. Divergent-thinking tasks are commonly used to estimate creative potential, but they do not represent creativity in its full developmental, social, and real-world forms (Runco & Acar, 2012). This evidence aligns with Leggett’s (2024) cross-cultural analysis, which highlighted that creativity in early childhood is best cultivated through open-ended and exploratory activities that support spontaneous thinking, rather than evaluations focused solely on final outcomes. Consequently, the results of this study are only applicable to figural divergent thinking and should not be generalized to other dimensions of creativity (such as verbal divergent thinking, social creativity, or actual creative behaviors in daily life).

Finally, the limitation in interpreting these findings is the reliance on teacher reports, which may introduce common method variance (CMV). The associations among creative personality dimensions and their parallel growth trajectories may be partially inflated by a halo effect in teacher perception. This teacher-rater bias offers a plausible alternative explanation for the high-order factor correlations. Subjective teacher expectations and the specific social dynamics of the classroom may foster a consistency bias, whereby preschoolers viewed as high-potential receive uniformly favorable ratings across all the creative traits. Nevertheless, in Study 3, teacher-reported tendencies were associated with preschoolers’ performance on an independent task-based measure of figural divergent thinking. This cross-method pattern reduces, but does not eliminate, the concern that the findings are only a product of teacher-rating bias. Crucially, these associations should not be taken as evidence for actual creative output or creative achievement more broadly.

## 6. Limitations

The current study still had the following limitations that need further exploration in the future.

First, preschoolers’ creative personality was assessed through teacher ratings. Teacher reports are useful in early childhood because teachers observe preschoolers across repeated classroom situations. However, these ratings also reflect what is visible and valued in classroom contexts. As discussed earlier, this introduces the potential for shared-method variance and the halo effect in teacher perceptions. Some preschoolers may show curiosity, independence, or sensitivity in ways that are less easily noticed by teachers. Therefore, the higher-order factors identified in this study should be interpreted as teacher-observed creative personality tendencies, rather than direct measures of preschoolers’ internal traits. Future studies should combine teacher ratings with parent reports, structured observations, and child-based tasks to provide a more complete assessment (De Los Reyes & Kazdin, 2005).

Second, the analysis was constrained by both factor selection and sample age demographics. Although a three-factor higher-order structure was suggested in Study 1, subsequent developmental analyses were restricted to the intrinsic driving and growing factors. While this decision was empirically necessary due to the factor’s low stability and the constraints of latent growth modeling in a modest sample, it means that the construct validity of the full three-factor model was not comprehensively tested over time. Consequently, the developmental and predictive conclusions are restricted to the core socio-motivational components of preschoolers’ creative personality. Furthermore, due to the uneven age distribution in Study 1, this imbalance may limit the factor stability across age groups. Therefore, developmental interpretations regarding the trajectory toward middle childhood should be made with caution, as the current model is heavily weighted by the 3–5-year-old majority. Future research utilizing larger, age-balanced samples and more refined measures is required to verify the invariance and longitudinal evolution of the complete three-factor structure.

Third, the longitudinal analysis in Study 2 was limited by both the number of measurement waves and the sample size. Although three annual waves are sufficient to identify linear growth, non-linear developmental patterns (e.g., quadratic growth) could not be evaluated. Thus, the findings primarily reflect a general maturational trend during the preschool years. Additionally, although the sample size is adequate for the simplified growth model, it remains limited for complex longitudinal structural equation modeling. Statistical power may be constrained when small effect sizes or complex model specifications (e.g., nonlinear trajectories or multivariate moderators) are evaluated. Therefore, the reported developmental trajectories should be interpreted with caution. To capture non-linear dynamics and confirm generalizability, larger sample sizes and more frequent assessments (e.g., four or more waves) should be utilized in future research.

Fourth, the samples were drawn from urban public preschool settings in China. This sampling context places clear limits on the generalizability of the findings. Public preschools in urban China may share relatively similar classroom routines, teacher expectations, curriculum arrangements, and evaluation norms. These features may shape both how preschoolers display curiosity, independence, cooperation, achievement, sensitivity, and novelty, and how teachers notice and rate these tendencies. Therefore, the present findings should not be read as representative of Chinese preschoolers as a whole, nor should they be generalized directly to rural preschools, private preschools, or early childhood settings in other cultural contexts. Future studies should test whether the present higher-order structure and its association with figural divergent thinking can be replicated across different regions, types of preschools, and cultural settings.

Finally, the evaluation of creative outcomes was constrained by a single-task and single-informant design. Measurement was restricted to a single figural divergent thinking task. Although robust, the TTCT represents a narrow operationalization of early childhood creativity, which inherently encompasses verbal, social, and motor domains. Thus, the observed predictive validity of the intrinsic driving factor might be specific to figural ideation. To address these limitations, a multi-informant and multi-task framework should be adopted in future research. Teacher and parent ratings, structured classroom observations, and a broader battery of creativity tasks (including verbal and play-based assessments) should be combined. Such comprehensive designs are required to verify whether the identified factors reflect stable, domain-general dispositions or merely represent context-specific, teacher-observed tendencies.

## 7. Conclusions

This study examined the higher-order structure of teacher-rated creative personality dimensions in preschoolers, their developmental trajectories, and their association with figural divergent thinking. In the present sample, exploratory factor analyses suggested that the nine teacher-rated dimensions could be represented by three higher-order groupings: intrinsic driving, growing, and openness. However, due to the longitudinal instability of the openness factor, the main longitudinal and predictive analyses focused on the two core factors. The intrinsic driving and growing factors showed parallel developmental increases, but the present data did not support directional relations between them. The intrinsic driving factor was more strongly associated with preschoolers’ performance on the Parallel Lines Task, whereas the growing factor showed a weaker and nonsignificant association in the structural model. These findings should be interpreted as evidence regarding teacher-observed personality-related tendencies and one specific form of figural divergent thinking, rather than as evidence for preschoolers’ creativity more broadly.

## Figures and Tables

**Table 1:** Participant information in Study 1 *M* (*SD*).

Age	Gender	N	Age (Years) *M* (*SD*)
3	Total	235	3.37 (0.47)
	Boys	117	3.35 (0.48)
	Girls	118	3.31 (0.46)
4	Total	268	4.41 (0.48)
	Boys	145	4.50 (0.50)
	Girls	123	4.32 (0.47)
5	Total	168	5.31 (0.47)
	Boys	86	5.39 (0.49)
	Girls	82	5.24 (0.46)
6	Total	30	6.18 (0.18)
	Boys	18	6.20 (0.20)
	Girls	12	6.15 (0.16)

**Table 2:** Factor model of preschoolers’ creative personality.

Model	Factor 1 (Intrinsic Driving Factor)	Factor 2 (Growing Factor)	Factor 3	Factor 4
Model 1	Curiosity, Sensitivity	Self-Confidence, Cooperation		
Model 2	Curiosity, Sensitivity, Novelty	Self-Confidence, Cooperation, Independence, Achievement		
Model 3	Curiosity, Sensitivity, Novelty	Self-Confidence, Cooperation, Independence, Achievement, Aesthetics, Sense of Humor		
Model 4	Curiosity, Sensitivity, Novelty, Aesthetics, Sense of Humor	Self-Confidence, Cooperation, Independence, Achievement		
Model 5	Curiosity, Sensitivity, Novelty	Self-Confidence, Cooperation, Independence, Achievement	Aesthetics, Sense of Humor	
Model 6	Curiosity, Sensitivity, Novelty	Self-Confidence, Cooperation, Independence, Achievement	Aesthetics	Sense of Humor

**Table 3:** Factor model fit indices.

Model	χ^2^	df	CFI	TLI	RMSEA (90% CI)	SRMR	AIC	BIC	LLH (H_0_)
Model 1	11.49	4	0.99	0.99	0.04 [0.00, 0.08]	0.04	34,673	34,754	−17,320
Model 2	14.24	13	0.99	0.99	0.02 [0.00, 0.05]	0.06	37,592	37,688	−18,777
Model 3	45.50	26	0.99	0.98	0.07 [0.04, 0.09]	0.06	55,340	55,466	−27,645
Model 4	43.15	26	0.99	0.99	0.06 [0.03, 0.09]	0.06	55,289	55,425	−27,617
Model 5	37.60	24	0.99	0.99	0.06 [0.03, 0.09]	0.05	55,200	55,310	−27,571
Model 6	267.42	36	0.75	0.67	0.20 [0.18, 0.23]	0.15	57,854	57,970	−28,904

**Table 4:** Participant information in Study 2 *M* (*SD*).

	T1		T2		T3	
	N	Age (Years) *M* (*SD*)	N	Age (Years) *M* (*SD*)	N	Age (Years) *M* (*SD*)
Total	160	3.36 (0.50)	148	4.37 (0.48)	142	5.38 (0.49)
Boys	84	3.41 (0.50)	75	4.38 (0.49)	74	5.39 (0.50)
Girls	76	3.31 (0.51)	73	4.35 (0.48)	68	5.36 (0.48)

**Table 5:** Descriptive statistics of preschoolers’ creative personality factors scores across three waves *M* (*SD*).

	T1		T2		T3	
	Intrinsic Driving Factor	Growing Factor	Intrinsic Driving Factor	Growing Factor	Intrinsic Driving Factor	Growing Factor
Boys	2.88 (0.68)	2.9 (0.66)	3.19 (0.60)	3.19(0.52)	3.26 (0.51)	3.28 (0.58)
Girls	2.83 (0.57)	2.98 (0.58)	3.23 (0.49)	3.34(0.46)	3.28 (0.47)	3.57 (0.43)
Total	2.85 (0.63)	2.93 (0.63)	3.20 (0.55)	3.26(0.49)	3.27 (0.48)	3.43 (0.53)

**Table 6:** Longitudinal Measurement Invariance for the Two-Factor Higher-Order Model Across Three Waves.

Model	χ^2^	df	CFI	TLI	RMSEA (90% CI)	SRMR	ΔCFI	ΔTLI
Configural	145.67	120	0.98	0.98	0.03 [0.01, 0.05]	0.03	——	——
Metric (loadings)	163.84	138	0.98	0.97	0.03 [0.01, 0.05]	0.03	−0.002	−0.002
Scalar (intercepts)	181.92	156	0.98	0.97	0.03 [0.01, 0.05]	0.03	−0.003	−0.003
Strict (residuals)	252.45	174	0.96	0.95	0.05 [0.04, 0.07]	0.08	−0.018	−0.018

Notes: Δ values are computed relative to the immediately preceding (less constrained) model. χ^2^ = chi-square; df = degrees of freedom; CFI = comparative fit index; TLI = Tucker–Lewis index; RMSEA = root mean square error of approximation; SRMR = standardized root mean square residual. —— = not applicable.

**Table 7:** Unconditional latent growth linear model fit indices.

	χ^2^	df	CFI	TLI	RMSEA (90% CI)	SRMR	R_T1_^2^	R_T2_^2^	R_T3_^2^
Intrinsic driving factor	4.26	3	0.98	0.97	0.05 [0.02, 0.08]	0.05	0.42	0.38	0.33
Growing factor	4.82	3	0.97	0.96	0.06 [0.03, 0.09]	0.06	0.48	0.33	0.35

**Table 8:** Parameter estimates of the unconditional latent growth linear model.

	Intrinsic Driving Factor		Growing Factor	
	Estimate (SE)	95% CI	Estimate (SE)	95% CI
Intercept Mean	2.89 *** (0.02)	[2.85, 2.93]	2.98 *** (0.02)	[2.94, 3.02]
Slope Mean	0.21 *** (0.02)	[0.17, 0.25]	0.21 *** (0.02)	[0.17, 0.25]
Intercept Variance	0.22 *** (0.04)	[0.14, 0.30]	0.09 *** (0.03)	[0.03, 0.15]
Slope Variance	0.05 * (0.02)	[0.01, 0.09]	0.01 (0.02)	[−0.03, 0.05]
Intercept–Slope Covariance	−0.03 * (0.02)	[−0.05, −0.01]	0.00 (0.01)	[−0.02, 0.02]
Intercept–Slope Correlation (*r*)	−0.31 ***	[−0.51, −0.11]	0.03	[−0.22, 0.28]

Note: *** *p* < 0.001, * *p* < 0.05.

**Table 9:** Estimated correlations between intrinsic driving factor and growing factor.

	*M* (SE)	1	2	3	4
1. Intrinsic driving factor *i*_1_	2.89 (0.06)	1			
2. Intrinsic driving factor *s*_1_	0.22 (0.04)	−0.26 ** [−0.41, −0.10]	1		
3. Growing factor *i*_2_	2.98 (0.05)	0.35 *** [0.20, 0.49]	−0.32 *** [−0.46, −0.16]	1	
4. Growing factor *s*_2_	0.23 (0.03)	−0.28 *** [−0.43, −0.12]	0.30 *** [0.14, 0.44]	0.15 [−0.02, 0.31]	1

Note: *** *p* < 0.001, ** *p* < 0.01. [ ] contains the 95% confidence interval.

**Table 10:** Descriptive statistics of preschoolers’ creative thinking.

	Age (Years)	Total (N = 170)		Boys (n = 91)		Girls (n = 79)	
		*M*	*SD*	*M*	*SD*	*M*	*SD*
Fluency	4 (n = 47)	3.03	3.42	2.26	2.48	3.77	4.05
	5 (n = 84)	5.96	3.79	5.30	3.63	6.73	3.88
	6 (n = 39)	6.76	4.75	7.24	5.27	6.06	3.94
	Total	5.34	4.18	5.02	4.24	5.70	4.11
Flexibility	4 (n = 47)	2.67	2.67	2.09	2.24	3.25	3.10
	5 (n = 84)	4.92	3.24	4.41	3.10	5.44	3.38
	6 (n = 39)	5.81	4.15	5.96	4.72	5.66	3.57
	Total	4.41	3.54	4.21	3.65	4.61	3.43
Originality	4 (n = 47)	5.21	5.41	4.51	4.60	5.88	6.18
	5 (n = 84)	8.11	6.02	7.42	6.04	8.90	6.58
	6 (n = 39)	7.53	6.94	7.48	7.18	7.59	6.60
	Total	7.17	6.93	6.70	6.27	7.72	6.52

**Table 11:** Correlation matrix between creative thinking and creative personality in preschoolers.

	1	2	3	4	5
1. Fluency	1				
2. Flexibility	0.93 ** [0.90, 0.95]	1			
3. Originality	0.88 ** [0.84, 0.91]	0.86 ** [0.82, 0.89]	1		
4. Intrinsic driving factor	0.30 ** [0.16, 0.43]	0.32 *** [0.18, 0.45]	0.20 * [0.05, 0.34]	1	
5. Growing factor	0.16 [−0.01, 0.31]	0.24 ** [0.09, 0.38]	0.07 [−0.08, 0.22]	0.66 *** [0.57, 0.74]	1

Note: *** *p* < 0.001, ** *p* < 0.01, * *p* < 0.05. [ ] contains the 95% confidence interval.

## Data Availability

The raw data supporting the conclusions of this article will be made available by the authors on request.
